# A comparison of survivourship and function (grazing and behaviour) of three gastropod species used as clean-up crew for the marine aquarium trade

**DOI:** 10.1371/journal.pone.0199516

**Published:** 2018-06-28

**Authors:** Gordon Watson, Jonathan Davies, Harriet Wood, Aleks Cocks

**Affiliations:** 1 Institute of Marine Sciences, School of Biological Sciences, University of Portsmouth, Ferry Road, Portsmouth, United Kingdom; 2 National Museum Cardiff, Cathays Park, Cardiff, United Kingdom; Czech University of Life Sciences Prague, CZECH REPUBLIC

## Abstract

Several million gastropods are collected each year for the marine ornamental trade to graze on algae detrimental to aquarium species, however, little is known about popular species’ suitability to perform this clean-up crew role. Three commonly traded gastropods, *Turbo bruneus*, *Tectus fenestratus*, and *Tegula eiseni* were assessed on their performance. Their survival was quantified as was their movement, and positioning with respect to water level and growth rates were calculated from the start and end weights. Nitrocellulose-coated slides were impregnated with an algal extract and the amount of grazing by each species was also compared. After 53 days final mortality levels of species were significantly different with all *T*. *bruneus* individuals surviving, whilst all *T*. *fenestratus* individuals apart from two and 35% of the *T*. *eiseni* had died by the end of the experiment. *T*. *bruneus* grazed significantly more than individuals of *T*. *eiseni*, and *T*. *fenestratus*. Both *T*. *bruneus* and *T*. *eiseni* were heavier after one month with *T*. *bruneus* gaining significantly more weight than *T*. *eiseni*. Greater percentages of algae were grazed by *T*. *bruneus* of increasing weight, although this relationship was not found for *T*. *eiseni* and *T*. *fenestratus*. All three species were generally active and remained within the water for the vast majority of time, although a small, but significant amount of time was spent out of the water for *T*. *eiseni*. *T*. *fenestratus* were significantly less active than *T*. *bruneus*, although the mean activity of *T*. *eiseni* was not significantly different from either species. *T*. *bruneus* out performs the other two species as a cleaning organism especially in the context of fluctuating water quality, thus highlighting the varying suitability of organisms for this task. Preference in the ornamental trade should be given to *T*. *bruneus* over the other topshells, but accurate species identification is critical.

## Introduction

The trade in marine ornamentals has grown dramatically in recent years with over 2 million people worldwide owning aquariums and worth in excess of US$200–330 million annually [1–4]. Advances in technology, husbandry techniques and increases in disposable incomes have enhanced a purchaser's ability to provide a home for these exotic organisms [5]. This has led to at least 10 million invertebrates (excluding corals) being collected per year [4,6,7], although recent analysis by Murray et al. [8] of traded polychaetes would suggest that this number is likely to be a 10–20 fold underestimate.

Not all of the organisms that feature in the trade are marketed for their aesthetic value, as some also have a practical use [9]. The health of many desirable organisms such as corals can be hindered by the presence of algae which can overgrow them and compete for light [10]. As a consequence, many gastropods from several families (e.g. Trochidae, Neritidae, Cerithiidae, Strombidae, Cypraeidae, Buccinidae and Nassariidae) are traded due to their perceived ability to perform a cleaning function within aquariums (marine and freshwater) by removing undesirable biotic matter including algae and diatom biofilms [11–13]. The benefit of having such organisms was demonstrated by Toh et al. [10] who showed that the presence of *Trochus maculatus* transplanted into an *ex-situ* environment improved the health of young *Pocillopora damicornis* corals leading to greater growth. Similarly, Villanueva et al. [12] have shown that *Trochus niloticus*' algal grazing has the ability to increase the survivorship of coral spat.

Whilst the price of gastropods in the trade is generally low, multiple individuals are often needed so as to perform the cleaning task to satisfactory levels within an aquarium [14]. It is this necessary clean-up crew function performed by many snails within one reef aquarium that has led to an increase in collection rates with grazer landings increasing by 26.4% per year from 1994 to 2007 in the Florida fishery. In 2007 this was equivalent to over 6 million grazers being collected and included 2.528 million gastropods [4]. Although evidence has not been gathered for many aquarium species, over collection from marine habitats can have major negative effects on the natural environment [15,16].

A variety of organisms are traded for their ability to graze in marine aquarium environments, although the most desirable organisms belong to the *Trochus*, *Turbo*, *Tectus*, *Tegula* and *Astraea* genera [9]. The species within the first three genera generally originate from shallow tropical reef waters [3]. Not all species, however, are not confined to tropical regions as Abbot [17] observed that the trochid *Margarites pupillus* can frequently be found along sub–tropical coastlines between the Bering Sea and San Diego.

**Fig 1:**
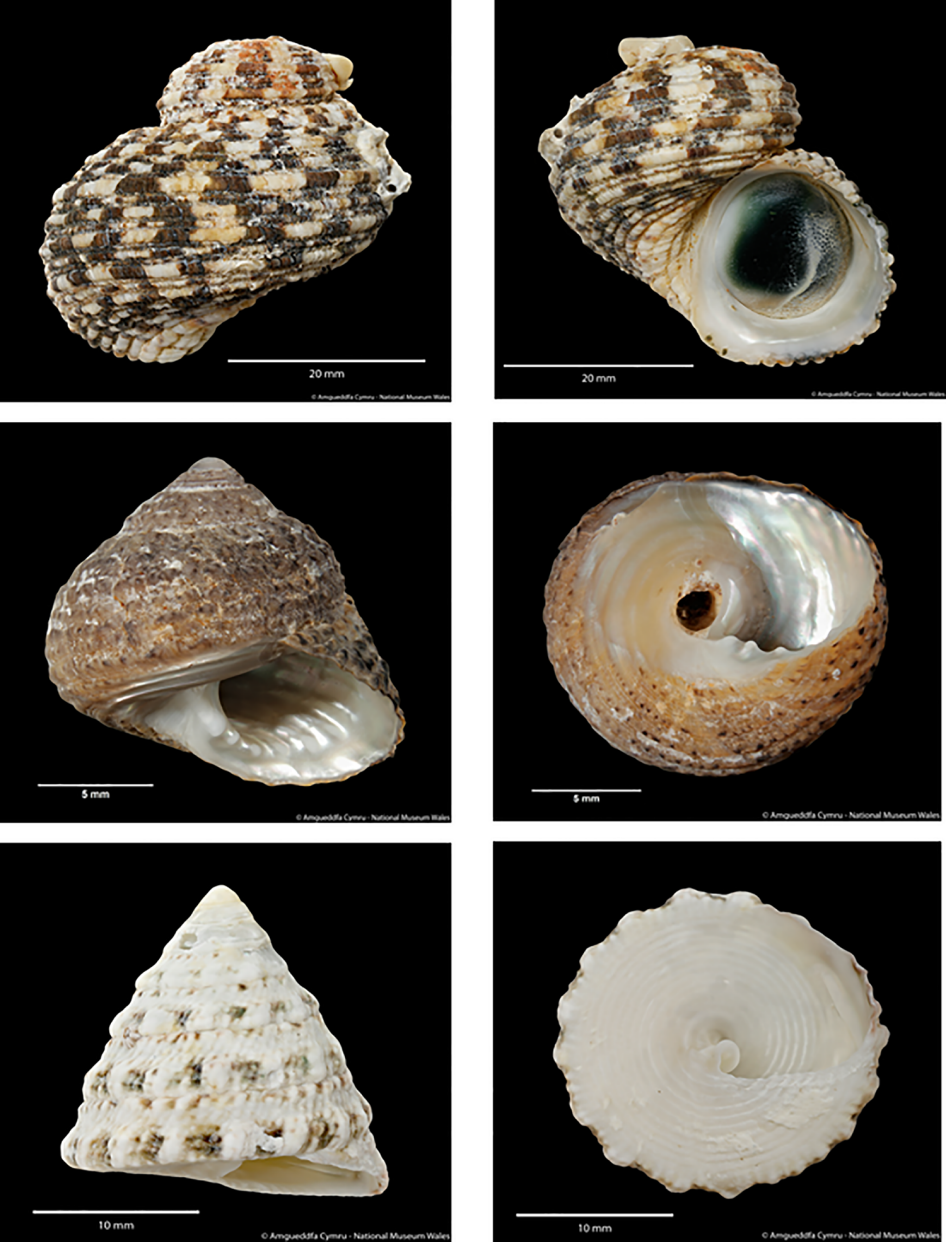
**Images of the three tropical gastropod species.** Top: *Turbo bruneus* (apex missing due to damage), Middle: *Tegula eiseni*, Bottom: *Tectus fenestratus*.

In order to be effective clean-up crew, Watson et al. [18] stated that snails must meet three key criteria, including not only staying alive, but also maintaining the ability to graze in these *ex-situ* conditions, and showing high activity while remaining below the waterline. As Alfaro et al. [19] highlight, there can be high variability in consumption rates between organisms even though many species have been traded for decades and the composition of herbivorous species occurring in a closed system has an important effect on how much grazing occurs [20]. Nevertheless, the strength of herbivory by gastropods for the aquarium trade remains scientifically unassessed [21]. Not only is this critical for choosing species as clean-up crew it is also important in an ecological context for coral reefs. The aim of this study is to determine the suitability of three commonly traded marine gastropods, *Tectus fenestratus* (Gmelin, 1791), *Turbo bruneus* (Röding, 1791), and *Tegula eiseni* (Jordan, 1936) as clean-up crew (Fig 1). To do this, the mortality and growth of these species over time was recorded; the level of algal grazing and activity levels quantified; and aquarium position recorded to assess species-specific behaviours and if consumption is related to size of the snail.

## Materials and methods

### Collection, maintenance and acclimation

Twenty snails of each species were purchased from a wholesaler and divided evenly among ten glass aquariums (30 x 30 x 34 cm) and labelled with Bee-tags (Abelo's Beekeeping Supplies) attached to their shells. Aquariums were filled with filtered (polywool) water from Langstone Harbour, Portsmouth, UK and were fitted with an internal biological canister filter, a heater set to 25°C and then covered with a plastic lid drilled with holes. Prior to the introduction of the snails, fishless cycling was performed to allow the establishment of the filter bacteria with snails only being added after ammonia and nitrite had fallen to zero. Physiochemical parameters (salinity, temperature and pH) were recorded using standard digital probes. Total ammonia (ammoniacal nitrogen), nitrite and nitrate were measured using a PalinTest photometer 7000se (Gateshead, UK). All parameters were measured two or three times a week (between 09:00 and 17:00) with nitrate levels recorded weekly. Food was provided in the form of three stones (approximately 5 cm in diameter) per tank each with 3g (wet weight) of the green alga *Ulva lactuca*, collected from Langstone Harbour, wrapped around them. These were replaced twice a week during tank maintenance and cleaning.

### Survivorship

Three stages of survivorship were used based on [22]. a) active: individual attached to the substrate by its foot, b) in-active: individual not attached to the substrate, but with foot moving after being prodded with a metal seeker, c) dead: individual showing no signs of movement of exposed foot and no response to seeker. Each individual’s state was recorded five times per week with dead individuals removed. Snails were also weighed at the start of the experiment and reweighed at one and two months and at the end of the experiment (after 53 days).

### Behaviour and activity

The position of each individual within the tanks was measured once a day (between 11:00 and 13:00) five days a week for four weeks by recording each snail’s location on the lid, base or a side and then converting to an activity score for each 24 hour period as described in Watson et al. [18]. Briefly, an individual remaining on (or returning to) the same face would score zero, whilst one that had moved to an adjoining face would score one, and two if it moved to an opposite face. The position of the individual relative to the air-water interface was also recorded as: fully submerged; fully out of the water; or at the air-water interface (defined as when some part of the shell broke the surface).

### Algae grazing

To quantify the process of grazing we used the method of Watson et al. [18] originally adapted from Farrell [23]. Briefly, a 4 cm diameter circle was scored on a 5 x 5 cm nitrocellulose-coated (0.1 mm layer thickness) glass or plastic base with all nitrocellulose outside of this area removed. An extract was then prepared using fresh fronds of *U*. *lactuca* (2 g blotted wet weight) ground for five minutes in a pestle and mortar with 1 ml of sea water and 0.06 g carborandum powder to produce a paste, and then diluted with 10 ml of sea water before centrifugation at 4000 rpm for three minutes at 20˚C. The supernatant was removed and 80 μl (enough to saturate the nitrocellulose) was pipetted onto the centre of each tile and dried for two hours before being placed in a beaker. Beakers were then randomly placed into five additional tanks measuring (35 x 55 x 21 cm). Grazing tanks were filled with local water from Langstone Harbour, and fitted with a pump and a heater set to 25˚C, in order to replicate the ten holding tanks. One individual snail was randomly placed in each 500 ml beaker (apart from control beakers, which aside from the glass slides were left empty). Each beaker was covered with a perspex sheet with holes drilled in, to allow water exchange and to prevent snails from climbing out. After 24 hours each glass slide was removed from the tanks and placed on a dark background. Images were taken with a Nikon D50 SLR camera and ImageJ photo-editing software was then used to crop the nitrocellulose coated circles in each photograph before the area grazed/lost was measured. Percentage loss of nitrocellulose was calculated by producing a binary image from a grey scale image with a manual threshold setting of 1–141. The salinity, temperature and pH of the water and levels of ammonia and nitrite in the grazing tanks were measured following the removal of individuals at the conclusion of each feeding experiment (one feeding experiment with all individuals was performed each week and repeated for four weeks). Nitrate levels were measured weekly with 50% water changes performed weekly on each tank.

## Ethical statement

This study was performed under the University of Portsmouth’s animal welfare and ethical review body—Application for approval of work involving animals, but not regulated by the Animals (Scientific Procedures) Act and was approved.

### Statistical analysis

Data were analysed using Minitab (V17) and all data were normally distributed with equal variances except physiochemical parameter data (even after transformations). Combined with a repeated measures experimental design of the ten holding tanks, these data were, therefore, ranked and then analysed using GLMs (General Linear Models) with tank and time as factors. To investigate if there were relationships between water quality parameters and mortality and species’ activity levels, Pearson correlations were performed. The final percentage mortality for the survivorship of each species was also analysed using GLMs on ranked data with species and tank as factors followed by Tukey’s pairwise comparisons. Differences in growth rates between species (*T*. *bruneus* and *T*. *eiseni* only) in terms of percentage weight gain were analysed using a Mann-Whitney U test of medians. The daily recordings of the position of individuals in relation to the air-water interface were averaged and then the ranked means of the percentage time spent out of the water per snail were analysed using a GLM with species and tank as factors with an associated interaction term included in the model followed by Tukey’s pairwise comparisons. Mean activity scores for each individual were averaged and then analysed using GLM as above, but without the interaction term.

For grazing, to avoid issues of variance suppression [24] the background loss of nitrocellulose in control beakers was included as a treatment and then compared with grazing from the other beakers (containing snails). Ranked mean (weeks combined) percentage values were then analysed using GLM with species and tank as factors. Water quality of the holding tanks and grazing relationships of all three species were investigated with Pearson correlations on all species combined and individually. Physiochemical measurements of the water from the tanks which held the beakers were analysed using a GLM on ranked data followed by Tukey’s pairwise multiple comparisons. Any relationships between mass of the snail (*T*. *bruneus* and *T*. *eiseni*) and grazing were explored using Pearson correlations.

## Results

### Environmental parameters in holding tanks

The water conditions in each tank (means, ranges and standard deviations of the means) are presented in Table 1.

**Table 1:** Physiochemical measurements for the holding and grazing tanks.

**Main tanks**			**Statistical results**	
**Measure**	**Mean (±SEM)**	**Range**	**Tank**	**Time**
Temp (°C)	24.92 ± 0.02	24.5–25.5	F_9,239_ = 26.4, p< 0.001	F_23,239_ = 0.8 p = 0.736
Salinity (psu)	35.16 ± 0.02	34.2–36.1	F_9,239_ = 12.5, p< 0.001	F_23,239_ = 23.3, p< 0.001
pH	7.76 ± 0.01	7.4–8.2	F_9,239_ = 10.6, p< 0.001	F_23,239_ = 8.4, p< 0.001
Ammonia (mg l^-1^)	0.016 ± 0.003	0–0.25	F_9,239_ = 2.7, p = 0.005	F_23,239_ = 4.5, p< 0.001
Nitrite (mg l^-1^)	0.51 ± 0.09	0–5	F_9,239_ = 2.38, p = 0.014	F_23,239_ = 8.8, p< 0.001
Nitrate (mg l^-1^)	6.69 ± 1.2	0–80	F_9,79_ = 1.09, p = 0.379	F_23,79_ = 25.1, p< 0.001
**Grazing tanks**			**Statistical results**	
**Measure**	**Mean (±SEM)**	**Range**	**Tank**	**Time**
Temp (°C)	24.9± 0.06	24.5–25.5	F_4,39_ = 33.6, p< 0.001	F_7,39_ = 1.0, p = 0.463
Salinity (psu)	35.6 ± 0.06	34.8–36	F_4,39_ = 2.9, p = 0.038	F_7,39_ = 14.0, p< 0.001
pH	8.2 ± 0.00	8.2	-	-
Ammonia (mg l^-1^)	0.013 ± 0.01	0–0.25	F_4,39_ = 2.3, p = 0.08	F_7,39_ = 1.0, p = 0.452
Nitrite (mg l^-1^)	0.06 ± 0.02	0–0.25	F_4,39_ = 1.0, p = 0.424	F_7,39_ = 35.3, p< 0.001
Nitrate (mg l^-1^)	0	0	-	-

Physiochemical measurements for the ten holding tanks used for survivorship, activity and tank positioning experiments and of the five tanks used for grazing experiments. Data are presented as means (± SEM) and ranges for total ammonia (mg l^-1^), nitrite (mg l^-1^), nitrate (mg l^-1^), pH levels, salinity (psu) and temperature (°C). Ammonia, nitrite, pH levels, salinity and temperature were measured three times a week, nitrate concentrations were measured weekly.

Although these means, associated SEMs and ranges show that the water parameters remained relatively constant over the experimental period, all parameters except nitrate (over time) and temperature (tanks) were significantly different among tanks and over time using ranked data. Pearson correlations confirmed that the only significant relationship for tank conditions was concentration of nitrite and mortality level (0.667, p = 0.035). However, for time there were significant negative relationships between mortality and nitrite (-0.867, p<0.001) and nitrate (-0.827, p = 0.011), but also significant positive relationships between mortality and pH (0.555, p<0.001) and temperature (0.414, p = 0.044).

### Mortality

**Fig 2:**
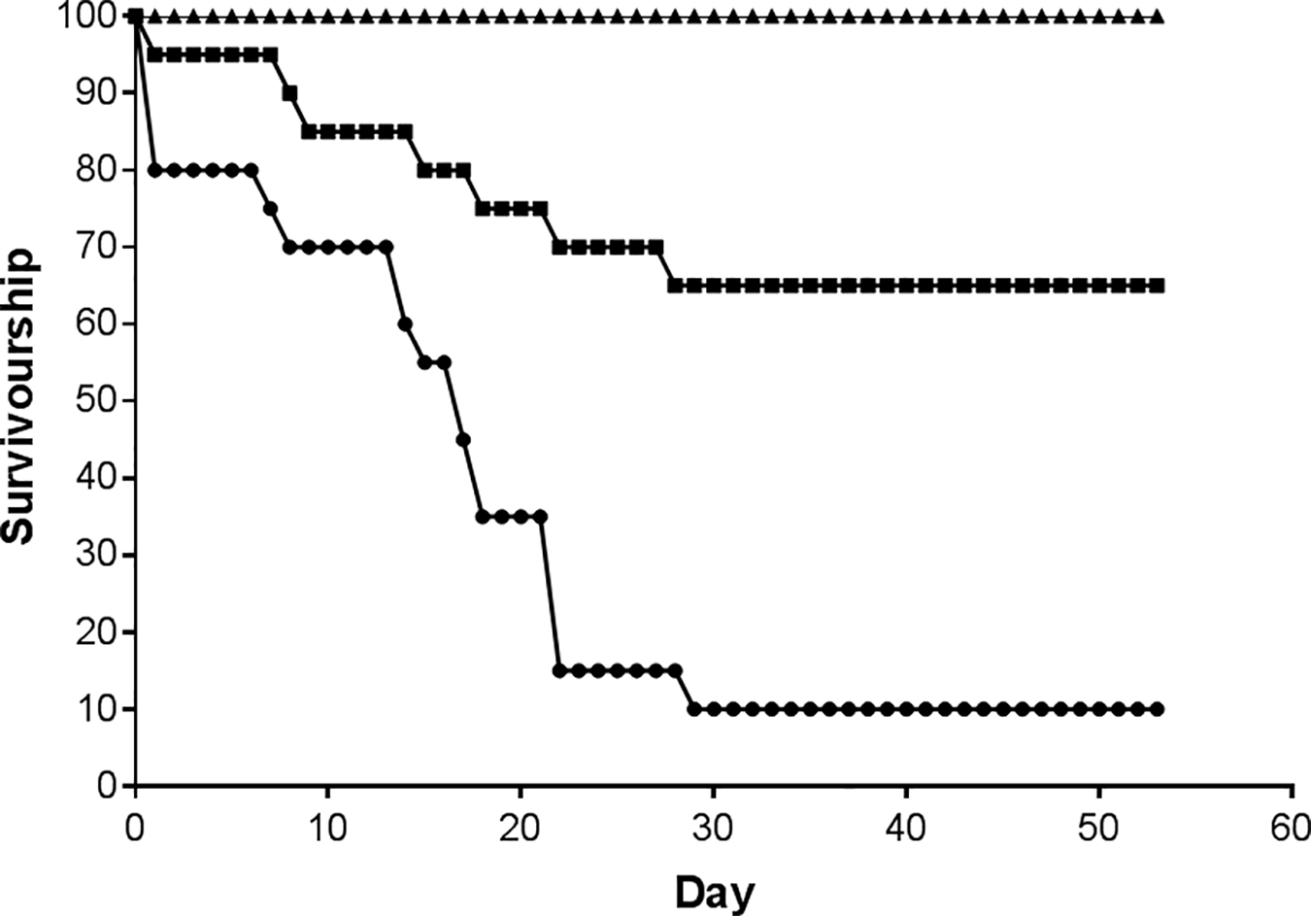
Number of living individuals of each species over 53 days. Filled triangle: *Turbo bruneus*, filled square: *Tegula eiseni*, filled circle: *Tectus*. *fenestratus*. 20 individuals of each species split across 10 tanks.

As shown in Fig 2, mean cumulative mortality varied dramatically among species with all of the *T*. *bruneus* surviving over the 53 days. In contrast, *T*. *fenestratus* mortality rate remained fairly constant for approximately 15 days, but all individuals apart from two had died by the end of the experiment. *T*. *eiseni* died at a rate of about one to two per week for the first four weeks, but with no further mortalities. Analysis of the ranked final mean mortality per tank confirmed that there were no significant differences among tanks, but highly significant differences among all species (GLM, F_2,29_ = 25.3, p<0.001) and when analysed using pairwise comparisons each species was different from the other. Inter-individual weight differences may have contributed to this variation in tolerance, however, there was no significant relationship between starting weight of an individual and the time it took to die for *T*. *eiseni*. In contrast, there was a significant, but negative relationship for *T*. *fenestratus* (r = -0.663, p = 0.005).

As food was provided throughout the experiment it was expected that the snails would gain weight and this was confirmed for *T*. *eiseni* and *T*. *bruneus* by analysing percentage weight gain (final wet weight compared to starting weight). *T*. *bruneus* individuals gained on average 34% ± 6.25 (n = 20) of their original starting weight, which was significantly higher than *T*. *eiseni* (13% ± 2.74, n = 13) and this is confirmed by a Mann-Whitney U test (W = 151, p = 0.01). It was not possible to assess weight gain for *T*. *fenestratus* due to the high levels of mortality.

### Behaviour and activity

**Fig 3:**
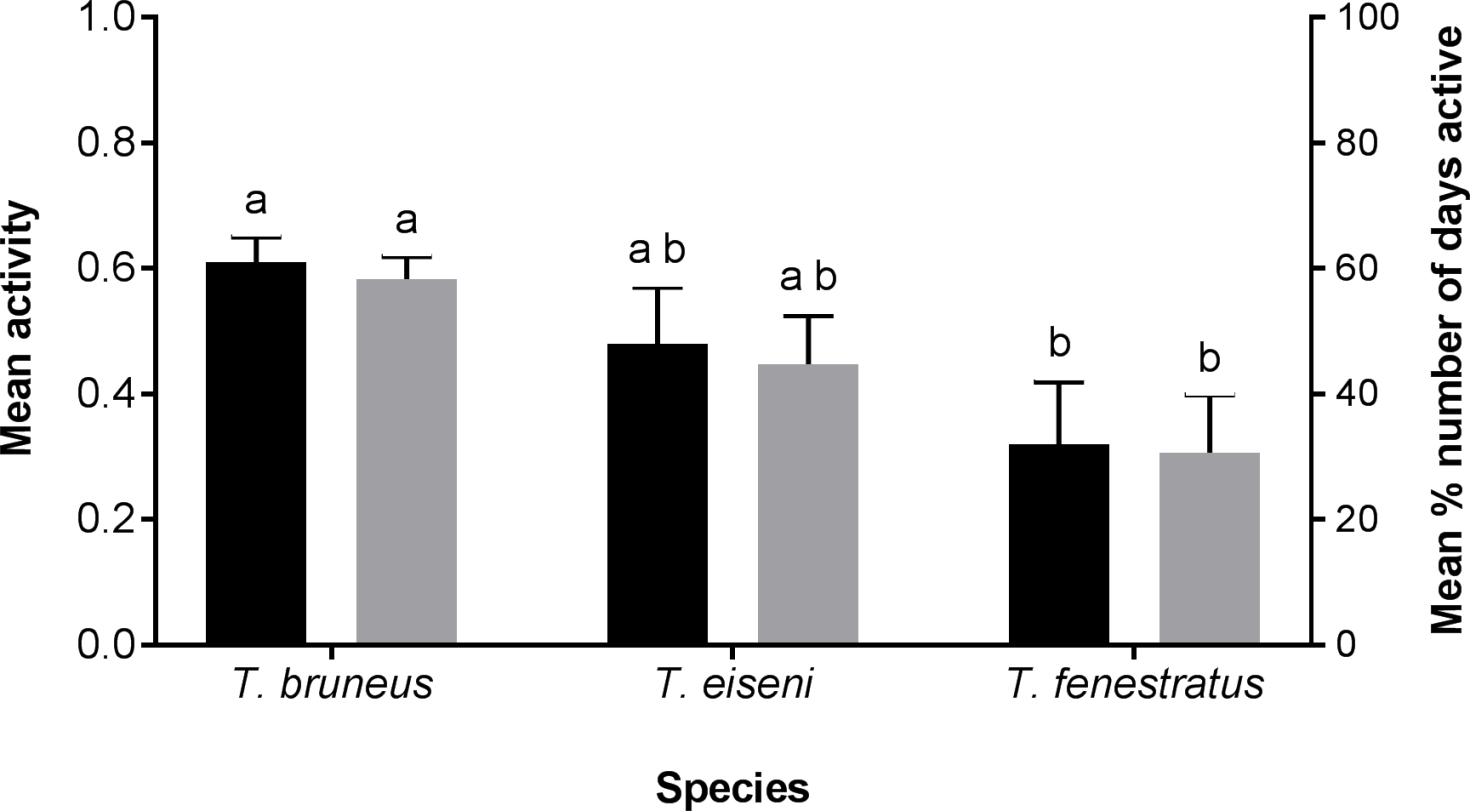
Mean activity score (± SEM) per individual (black bars) and mean percentage number of days active (± SEM) per individual (grey bars) for *Turbo bruneus*, *Tegula eiseni and Tectus fenestratus*. See text for explanation of activity calculation. N = 2 individuals of each species per tank, 10 tanks in total.

**Fig 4:**
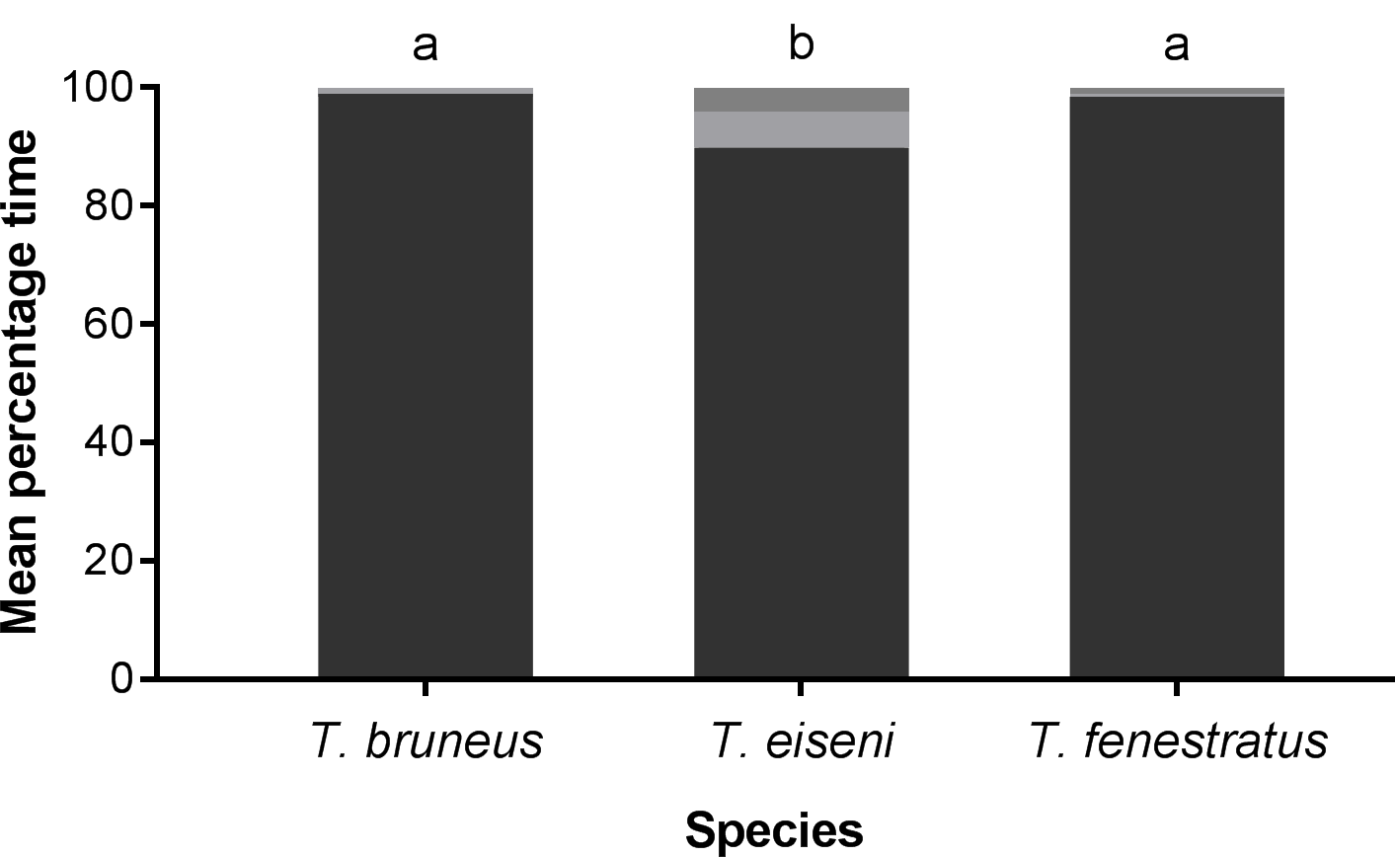
Mean amount of time (SEM not shown for clarity) *Turbo bruneus*, *Tegula eiseni and Tectus fenestratus* individuals spent at specific positions in relation to air/water interface. Submerged (black): individuals fully submerged; Out of water (light grey): fully out of the water; Air/water interface (dark grey): some part of the shell has broken the surface. N = 2 individuals of each species per tank, 10 tanks in total.

All species were generally active throughout the experiment with mean activity scores (a proxy for snail movement) above zero (Fig 3). All but six individuals (four *T*. *fenestratus* and two *T*. *eiseni*) were recorded as having locomotor activity: i.e. their recorded position changed. However, the mean activity score per individual confirmed that differences in activity level were present among species (F_2,29_ = 3.28, p = 0.048), but with no difference among tanks. Multiple comparisons confirmed that *T*. *fenestratus* individuals were significantly less active than *T*. *bruneus*, although the activity of *T*. *eiseni* was not significantly different from either species. These results were also repeated for the mean percentage number of days each snail was active for (GLM ranked percentages F_2,50_ = 3.71, p = 0.021, no difference among tanks) (Fig 3). Analysis of the mean percentage time spent out the water per individual (Fig 4) confirmed that for all species the vast majority of individuals were always submerged (means of 99% for *T*. *bruneus*, 89% for *T*. *eiseni* and 98% for *T*. *fenestratus*). Nevertheless, *T*. *eiseni* individuals spent a small, but significant proportion of their time at the air-water interface or out of the water compared to the other two species (GLM on ranked data F_2,53_ = 4.46, p = 0.023 with no difference among tanks). These results contrast with *Phorcus lineatus*, an inter-tidal temperate species, where individuals were less active and fully submerged for only 59% of the time (data not shown).

### Grazing

The mean temperature (±SEM) of sea water in the tanks used for the grazing was 24.9°C ± 0.05 (Table 1). Although there were significant differences among tanks (GLM of ranked data F_4,39_ = 32.29, p<0.001), the mean tank temperatures had a range of only 24.46–25.46°C. Concentrations of ammonia measured in four of the tanks were zero across all sampling points, whilst the concentration in the fifth was only a maximum of 0.25 mg l^-1^, therefore, leading to no significant differences among tanks or over time. This was also the case for nitrate and pH which both had constant values across tanks and time of 0 mg l^-1^ and 8.2, respectively. Salinity within the tanks varied significantly over time (GLM rank data F_4,39_ = 13.96, p<0.001) and among tanks (GLM ranked data F_7,39_ = 2.93, p = 0.038), but mean values per tank were within 0.3 to 1 psu of each other.

**Fig 5:**
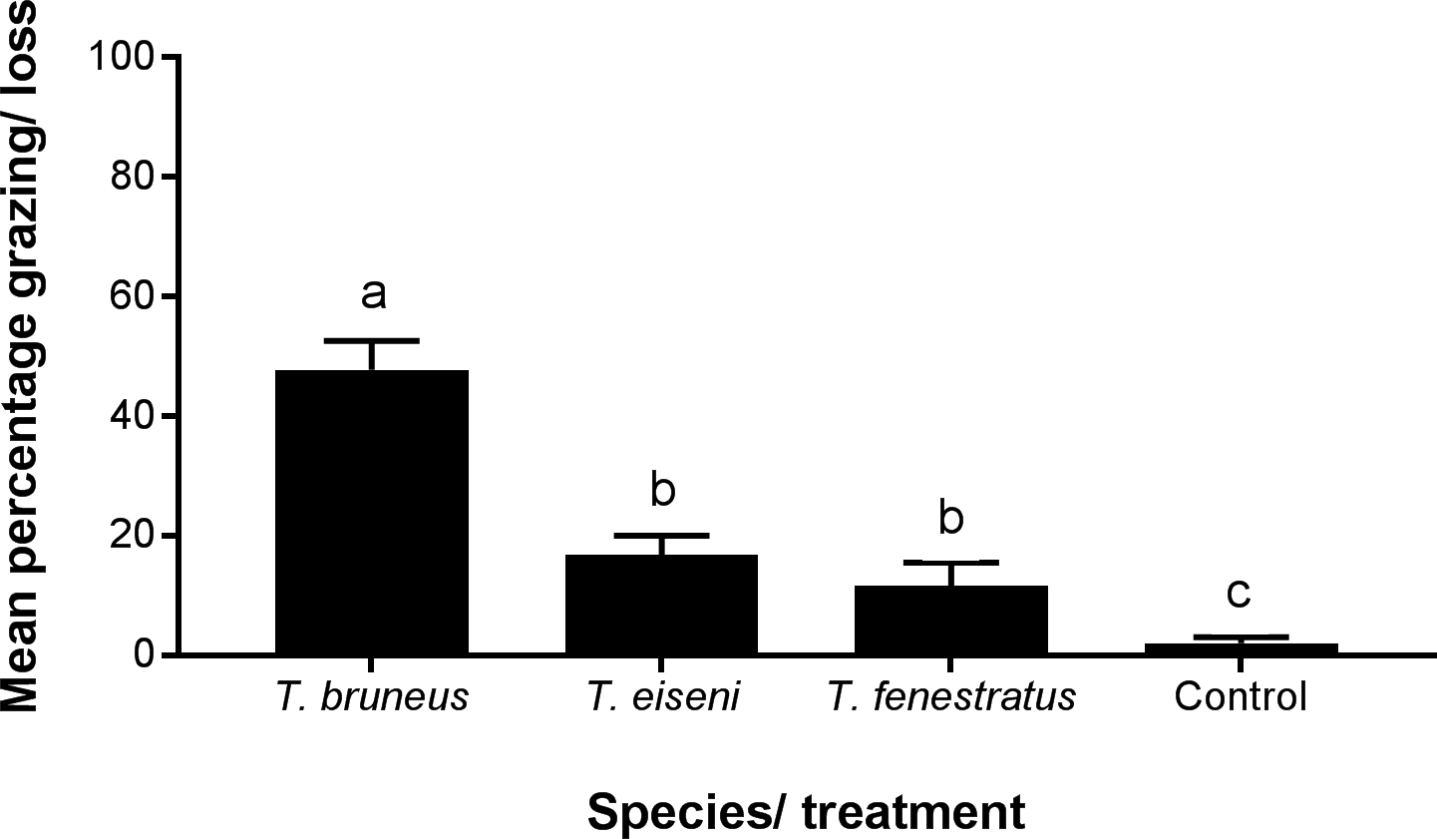
Mean percentage of nitrocellulose plate (± SEM) consumed/lost per individual for *Turbo bruneus*, *Tegula eiseni*,*Tectus fenestratus* and control after 24 hours. One individual per beaker (plus a beaker with no snail as a control) with 6 beakers per tank.

Observations of the plates after 24 hours of incubation with the snails confirmed that distinctive radula grazing patterns were present on many plates for all species. All nitrocellulose plates recorded a loss, however, wide variations occurred among individuals with some consuming 97% whilst others consumed very little. Mean percentage consumption data per species are presented in Fig 5 and confirmed that all species consumed nitrocellulose within 24 hours. Analysis shows that the level of consumption was different among species and the background loss (GLM rank data F_3,64_ = 53.5, p<0.001) and also across tanks (GLM rank data F_9,53_ = 2.6, p = 0.027). Further analysis with multiple comparisons showed that the background loss of nitrocellulose was significantly less than all three species, but *T*. *bruneus’* mean percentage consumption was significantly higher than the other two species, which were not significantly different from each other. The only significant relationship between tank conditions and grazing rates (all species combined and separate species) was the concentration of ammonia (0.732, p = 0.016), but this was positive i.e. an increase in ammonia was correlated with an increase in grazing.

**Fig 6:**
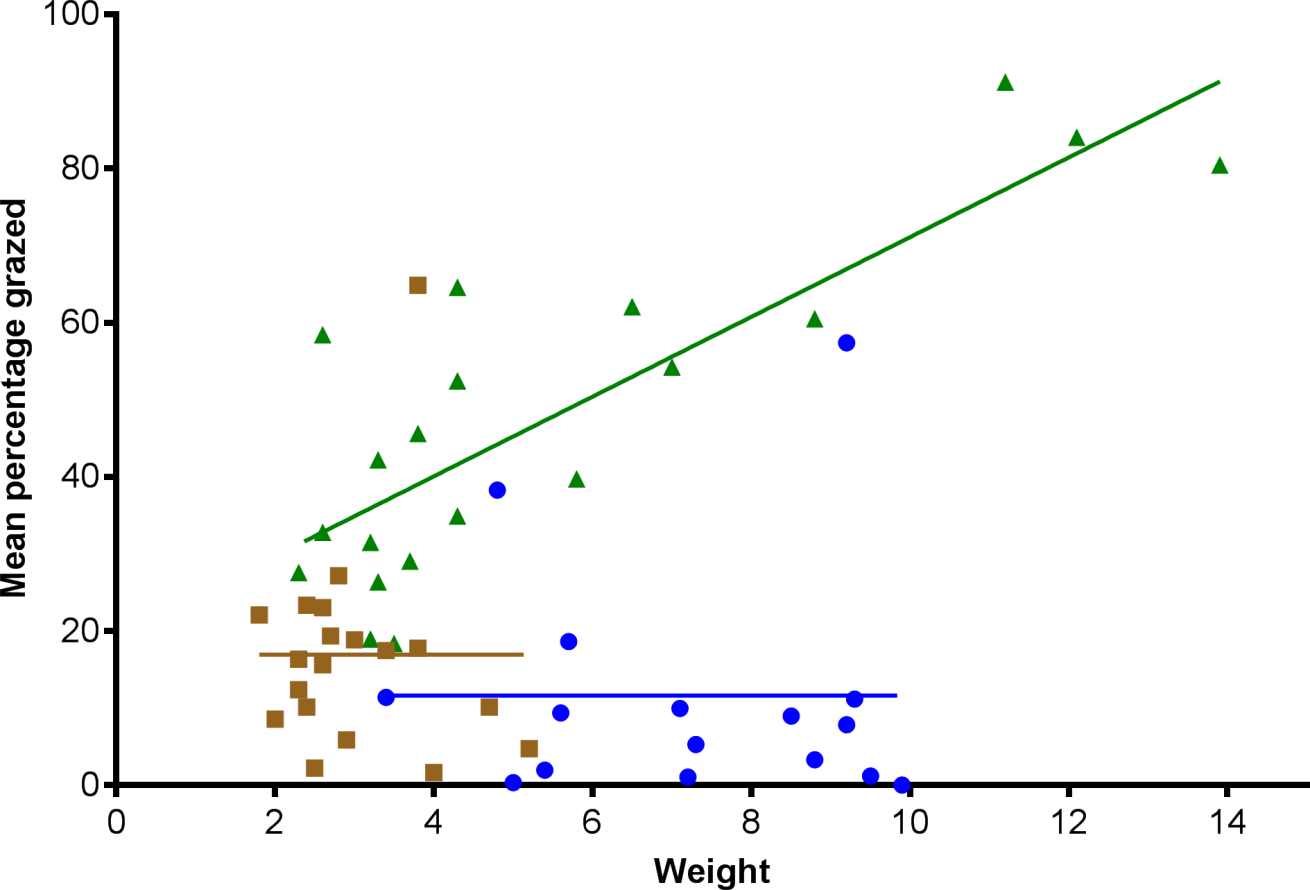
Relationship between wet weight (g) and percentage algae-impregnated nitrocellulose consumed per individual for *Turbo bruneus* (green triangle), *Tegula eiseni* (yellow square) *and Tectus fenestratus* (blue circle) after 24 hours. One individual per beaker (plus a beaker with no snail as a control); 6 beakers per tank with 5 tanks.

To see if the inter-individual differences in grazing were related to snail size Pearson correlations for each species were performed and start wet weights plotted against mean grazing rates with linear regression lines fitted (Fig 6). Although all species showed substantial variation in grazing, only *T*. *bruneus* had a strong positive correlation (r = 0.824) that was also highly significant (p<0.001).

## Discussion

### Mortality

Hobbyists’ experiences and observations over many years have supported the view that some marine invertebrate species are not suited to captivity as they do not survive for long periods of time in aquariums. Our data are the first to confirm that there is significant differential survival for gastropod clean-up species that are commonly traded, with *T*. *bruneus* clearly the most suitable species owing to the 100% survival. In contrast, *T*. *fenestratus* would be completely inappropriate with nearly all individuals dying rapidly. The mortality rate of *T*. *eiseni* was initially quite high, but did stabilise mid-way through the experimental period suggesting that certain individuals might be suited to an aquarium environment if given appropriate acclimation time. Inter-individual weight differences may have contributed to this variation in tolerance, however, only a significant, but negative relationship for *T*. *fenestratus* existed. This would indicate that the heavier animals are less tolerant, which is without clear explanation. Regardless of the presence or absence of differences in individual tolerance (and the underlying drivers of these), the level of mortality shown by these two species would be unacceptable for a hobbyist’s aquarium. Although the survivorship data for *T*. *bruneus* are excellent, results from other species in the genus would suggest that longer term experiments are still required. For example, Purcell et al. [25] found survivorship of *T*. *niloticus* fell to 36–47% over six months in captivity.

Water quality and associated environmental conditions are key to long term survival of an aquatic organism. Physiochemical parameters within the holding tanks remained relatively constant over the experimental period and were generally within acceptable limits. Nevertheless, there were significant differences between tanks and changes over time for all water quality parameters except nitrate (over time) and temperature (tanks). The significant relationships over time for nitrate and nitrite are unlikely to be drivers for mortality as they were negative (decreasing concentrations, but increasing mortality over time). For temperature, which was positive (increasing temperature correlated with increasing mortality), this parameter only varied by 0.23°C across sampling points. For pH, means of all holding tanks were below 8.0, and in marine gastropods, such as *Busycon canaliculatum*, an individual's oxygen affinity is reduced in a pH range of 6.6–7.9 [26], which may have contributed to the mortality. The significant correlation of nitrite and mortality among tanks indicates that nitrite could have contributed to the mortality of *T*. *fenestratus* and *T*. *eiseni* as elevated concentrations were recorded, with some tanks reaching 5 mg l^-1^. However, a number of tanks had means of between 0.07–0.09 mg l^-1^ over the 53 days and those that had substantially higher means did so because of elevated concentrations recorded on the first three to five sampling points only. Nearly all other subsequent sampling dates (19 in total) for all tanks had concentrations of either 0.25 or 0 mg l^-1^. These initial increases in nitrite probably resulted from the early mortality events within the tanks briefly overwhelming a biological filtration system that was not fully competent to convert nitrite to nitrate at that time. Nitrite is known to be toxic to invertebrates as it removes the ability of pigments to carry oxygen [27], as demonstrated by Cheng and Chen [28] through the inverse relationship between nitrite concentration and oxyhaemocyanin levels (oxygen carrier common to both arthropods and molluscs [29]). Increasing concentrations of nitrite have also been shown to lower the amount of food eaten in other gastropods such as the greenlip abalone *Haliotis laevigata* [30]. Nitrite tolerance is species dependent, with authors reporting high levels of survivorship in acute tests and high No Observed Effective Concentrations for a range of sub-lethal endpoints measured in some bivalves and crustaceans [31–33]. However, in an invertebrate reef aquarium even trace levels of nitrite are not desirable [34]. Further work is required to remove the influence of elevated nitrite and decreasing pH, but if they are a contributory factor it also highlights that *T*. *bruneus* is the most tolerant species to less-than-perfect water quality and, therefore, even more appropriate as aquarium clean-up crew.

Previous experiments have shown that competition for food can cause higher mortalities [35–37] and was suggested by Watson et al. [18] as a driver for differential species mortality in two temperate trochids. The potential explanation for differences in mortality here is unlikely to be food as it was provided in excess through regular replacement of the stones. In addition, both *T*. *bruneus* and *T*. *eiseni* gained significant body mass suggesting that individuals were consuming food. It was not possible to assess weight gain for *T*. *fenestratus* due to the high mortality so this species may have been outcompeted by the other two. This is supported by evidence that marine gastropods with a high aspect ratio (tall spine) often exhibit avoidance behaviour [38] resulting in *T*. *fenestratus* having reduced access to the macroalgae by avoiding the other species. This lack of competitive ‘aggression’ in this species is another reason for its unsuitability in a multi-species aquarium.

### Activity level

The fact that all three species had scores above zero and only six individuals did not move at all indicates that the species were active. Consequently, all three species meet the requirement of a clean-up crew organism to be active, although they were less active when compared to the inter-tidal *P*. *lineatus*. This may be due to the different experimental systems that were used, but relatively lower activity levels could have been due to other reasons. Firstly, climbing can be attributed to foraging behaviour [39]. As supplementary food was provided on the bottom of the tank individuals may have not needed to graze other areas of the tank; remaining on the bottom leading to a lower score with the chosen assessment method. Secondly, the lack of movement of *T*. *fenestratus* may be due its tendency to hide when threatened instead of moving large distances away from the threat or a competitor [38, 40]. Finally, the activity levels observed in this study, as well as the individual's positioning within the tanks could have been influenced by the sub-optimal water quality. Even though there were no significant correlations of activity level with any of the water parameters measured, Alonso and Camargo [41] showed that high nitrate levels can slow the velocity of the aquatic snail *Potamopyrgus antipodarum*.

*Turbo bruneus*, *T*. *eiseni* and *T*. *fenestratus* all meet the basic activity requirement and were nearly always submerged during the experiments. Both *T*. *bruneus* and *T*. *fenestratus* can be found in the inter-tidal region [42, 43], but unlike the inter-tidal *P*. *lineatus* (which spent over 40% of its time not submerged) the amount of time spent not-submerged was very low. *Tegula eiseni* did spend a small, but significant amount of time at the water/air interface or out of the water, but Richards et al. [44] only stated it to be a benthic species and did not confirm if it was found in the inter-tidal region. Regardless of the source habitat, this small amount of time spent out of the water may still be an issue for aquariums that do not have close fitting lids, resulting in loss of individuals as they crawl out of the aquarium completely.

### Grazing

All three tropical species consumed nitrocellulose in the grazing experiment (compared to the background losses) at levels similar to *Gibbula umbilicalis* and *P*. *lineatus* [18]. Results show that *T*. *bruneus* performed the most effectively; fulfilling the clean-up crew criteria by consuming significantly greater amounts than either of the other species. Across the species there was significant inter-individual variation in grazing rates which could be related to sub-optimal water quality of the holding tanks. However, there was only a significant and strong positive correlation between level of grazing of individuals for *T*. *fenestratus* and ammonia concentration. It is unclear why this relationship is positive considering the well-known toxicity of ammonia (e.g. [33]) and so requires further investigation.

Ng et al [45] showed that some gastropods exhibited a preference for a specific algal species so future studies should expand the type of algae tested to include some of the nuisance macroalgae such as *Caulerpa* spp., *Chaetomorpha* spp. and *Valonia* spp. [46] found in aquariums. Species selectivity could contribute to the differences in the consumption seen in this study, however, Foster and Hodgson [47] showed that other grazers such as *Turbo sarmaticus* can graze on a diverse range of algae including those from the Rhodophyta, Chlorophyta, and Phaeophyta. Assuming this broad diet is a common trait of species of the *Turbo* genus, it further supports *T*. *bruneus* as the ideal choice of clean-up crew.

The significant positive relationship between weight and consumption rate for *T*. *bruneus* is supported by a similar relationship for *P*. *lineatus* (data not shown) and other species (*T*. *sarmaticus*) in the field [47], although for some herbivorous molluscs lighter individuals have been noted to feed more quickly than heavier individuals [48]. Size-specific grazing rates of *T*. *bruneus* would enable hobbyists to size-match individuals with the level of algal removal needed in their aquarium, but this could also lead to over grazing within an aquarium as the snails grew. For *T*. *fenestratus* and some of the *T*. *eiseni* elevated mortality and poor condition could have dampened their feeding activity levels and would, therefore have masked any influence of size, although in other species no such relationship exists [6].

### Next steps for the hobby

It is important to note that specimen identification using [16, 49–52] occurred after the experiments had been performed, but what we originally requested from the suppliers were actually three other species: *T*. *fluctuosa*, *Astraea tecta* and *Margarites pupillus*. Even though these names are routinely used in the trade, only *M*. *pupillus* is an accepted taxon [53]. *Turbo fluctuosa* is considered to be a misspelling of *T*. *fluctuosus* [54], but for *A*. *tecta* this is not included in the WoRMs database, but one of the synonyms (*Trochus tectus)*, now known *as Lithopoma tectum* is [55]. Regardless of the correct nomenclature of the three requested species, it is clear that misidentification in the trade is a fundamental issue and has already been highlighted for other invertebrates [8]. Many gastropods are challenging to identify without specialist knowledge and sacrifice of the specimen so it is, maybe, unsurprising that the species delivered turned out to be different from requested. However, without accurate identification achieving sustainability is extremely challenging to do [56]. Considering the differing ability of the three species tested to meet the basic clean-up crew requirements [18] it is imperative that for candidate gastropods (and likely many other species) an assessment of the actual species traded is performed. This could then be used to generate an easy guide suitable for use within the trade and by the hobbyist to select only those species that are functionally/behaviourally suitable.

Michael [57] highlighted that whilst a species' ability to feed and endure an *ex-situ* environment's conditions are important, other factors such as the breadth of an individual's diet; its ability to compete for resources and to cope with sub optimum water quality should also be taken into consideration when evaluating a species' suitability for an aquarium. Our data have shown that *T*. *fenestratus* and to a lesser extent *T*. *eiseni* perform significant grazing under controlled conditions, but it is *T*. *bruneus* that can survive, grow most rapidly and function in what were sometimes sub-optimal water conditions, which as shown by the mortality was not possible for *T*. *eiseni*. This sets *T*. *bruneus* apart as the clean-up-crew of choice. Nevertheless substantial gaps remain in our understanding of cleaner-crew function in an aquarium setting. Amongst herbivorous invertebrates the general assumption is that algae availability is a limiting resource [58] leading to competition (within and among species), resulting in lowered growth rates, and reduced survivorship [59, 60]. Scientifically assessing how many clean-up crew (not just limited to gastropods) are needed to efficiently undertake the necessary clean-up roles within a specific aquarium volume and with known stocking levels and maintaining this activity over extended periods of time is also an essential next step. Such knowledge would underpin advice on how many are needed to fit an individual aquarium’s requirements. This would have important consequences for reducing excess purchasing and, ultimately lowering the number of individuals being collected from the wild.

## Conclusion

By virtue of *T*. *bruneus*' low mortality and relatively high grazing ability this species is more than capable of performing the role of a clean-up organism, and to a far greater extent than either *T*. *eiseni* and *T*. *fenestratus*. *Turbo bruneus* also remains active and spends the vast majority of its time below the waterline and grows rapidly. It, therefore, fulfils all of the criteria identified by [18] as being required of clean-up crew species. *Tegula eiseni* and *T*. *fenestratus* however, fulfil only two and would be of much lower value to hobbyists in terms of function. The lower survivorship of these two species in aquariums suggests that if these species are desired by hobbyists they would need to be repeatedly restocked; resulting in a much greater wild collection.

Clean-up species are perceived to be of lesser value than other traded aquatic animals as shown by their pricing structure, and as a consequence culturing these species is not a priority [61]. However, aquaculture methods have been developed for topshells (e.g. *T*. *niloticus*) which could be easily transferred and adapted to *T*. *bruneus* [62]. Nearly 20 years ago Foster and Hodgson [47] recommended the culture of turbinid gastropods due to their ability to withstand varying water temperatures, high tolerance for poor husbandry, and their capacity to eat large quantities of algae [63]; all qualities exhibited by *T*. *bruneus* in this study. Aquaculture is regarded as a key challenge and opportunity for the marine aquarium trade moving forwards and could alleviate some of the impacts of the trade [9]. Culturing *T*. *bruneus* would be an excellent exemplar of a ‘sustainable approach’, but crucially one that encompasses culture of *all* components of the marine livestock trade.
